# Anti-cancer Potential of Captopril and Botulinum Toxin Type-A and Associated p53 Gene Apototic Stimulating Activity

**DOI:** 10.22037/ijpr.2019.1100800

**Published:** 2019

**Authors:** Rania Ibrahim Shebl

**Affiliations:** *Department of Microbiology and Immunology, Faculty of Pharmacy, Ahram Canadian University (ACU), Cairo, Egypt.*

**Keywords:** Captopril, Botulinum toxin, Anti-cancer, Apoptosis, Metastasis

## Abstract

Mutational inactivation of p53 is a key player in the development of human cancer. Thus, retrieving the tumor suppressor activity of p53 gene is considered a novel strategy in cancer therapy. Current study aimed to investigate the anti-cancer potentials of botulinum toxin type-A (BTX-A) and captopril as a trial to shed light on effective anti-cancer therapy with lower side effects. Cytotoxic effect of captopril and BTX-A was determined using MTT assay against colon (HCT116) and prostate cancer (DU145) cells compared to their effect on normal vero cells. Anti-proliferation assay and anti-metastatic effect were carried out using trypan blue exclusion method and wound scratch migration test, respectively. The ability of test drugs to induce apoptosis in cancer cells was examined using real time PCR. Recorded data revealed that captopril exhibited a statistically significant cytotoxicity (*P* < 0.05) to cancer cells (IC_50_ values of 1.5 and 1.2 mg/mL) with much lower toxicity to normal cells. At the same time, IC_50_ values post BTX-A treatment were 7.2 and 6.4 U/mL for HCT116 and DU145 cells, respectively without any toxicity to vero cells. Both drugs showed inhibitory potentials on cellular proliferation and the ability of cancer cells to migrate in scratched monolayers was obviously inhibited along with increasing their concentrations. P53 expression levels in captopril and BTX-A treated DU145 cells were elevated by 4 and 2.5 folds, respectively, while lower level of apoptosis induction in HCT116 cells was observed. Accordingly, BTX-A and captopril could present potential anti-cancer candidates through triggering cancer cells towards self-destruction.

## Introduction

Cancer is considered a serious public health problem with increasing morbidity and mortality rates all over the world, thus there is an urgent demand for a more efficient treatment (1). Cancer therapy is still not successful for many patients in spite of many classical treatments available as chemotherapy, radiotherapy, and surgery. That is due to resistance, severe side effects caused by chemotherapy and radiotherapy, as well as their failure to prevent metastases. Accordingly, the use of complementary and alternative medicine in cancer treatment may present a new and effective therapy compared to conventional anti-cancer drugs (2). 

P53 is a tumor suppressor gene that plays a critical role in maintaining the genetic stability of somatic cells. High frequency of mutation in P53 gene that was reported in approximately half of all cancer cases counteracts its pro-apoptotic efficacy in preventing the proliferation of genetically altered cancer cells (3). In this context, reactivation of apoptosis through p53-dependent pathway is considered as an efficient approach in anticancer therapy (4). 

Biotoxins are considered a natural source of novel compounds that attracted the attention of many researchers to be an initial step in the design of new pharmacologically active compounds in cancer treatment (5). Studies have reported that snake and bee venom derived toxins as well as some bacterial and plant toxins could potentially aid in cancer therapy (1). Botulinum neurotoxin, which is produced by an anaerobic spore forming Gram-positive Clostridium botulinum*, *is one of these toxins. Botulinum neurotoxins are seven serotypes (from letter A to G) that are different in their antigenicity but showing the same pharmacological activity (6). All serotypes of botulinum toxin interfere with neural transmission at the neuromuscular junction through inhibiting the release of acetylcholine from the nerve ending resulting in muscle paralysis. Botulinum toxin is also known as miracle poison as it plays an important role in the cure of various clinical cases especially strabismus, focal dystonias, hemifacial spasm, spastic movement disorders, headaches, hypersalivation, correction of wrinkles, anal fissures, and hyperhidrosis. Botulinum toxin type A is the only commercially available serotype and was FDA approved in 2002 as Botox for the treatment of wrinkles resulting from facial expressions but its effect terminates after about three months. It was also reported that botulinum neurotoxin could facilitate the damage of cancer cells through opening tumor vessels thus allowing radiotherapy and chemotherapy to reach the cancer cells (7). 

Captopril is an angiotensin-I-converting enzyme (ACE) inhibitor that was approved as antihypertensive drug by FDA in 1981 (8). Captopril is currently effective in the treatment of cardiovascular diseases such as congestive heart failure and arterial hypertension (9). It was reported that patients treated with ACE inhibitors were at lower incidence of developing cancer (10). As a consequence, previously reported findings attracted our attention to investigate the cytotoxic and the apoptotic stimulating potentials of both BTX-A and captopril against cancer cell lines *in-vitro.*

## Experimental

*Materials*

Captopril was kindly supplied from Sedico Company-Egypt; botulinum toxin type A was purchased as Botox from Allergan-Ireland (100 U/vial).

Colorectal carcinoma cells; HCT116 cells (ATCC-CCL 247), human prostate cancer cells; DU145 cells (ATCC-HTB 81) and African green monkey kidney cells; vero cells (ATCC-CCL-81) were kindly supplied from the Egyptian company for biological products and vaccines (VACSERA), cell culture Department-Egypt. 

*Methods*

*Determination of cytotoxicity and morphological changes*

Cytotoxic effect of captopril and botulinum toxin type A (BTX-A) was evaluated against human cancer cell lines (DU145 and HCT116) as well as normal (vero) cells using 3-(4,5-dimethylthiazol-2-yl)-2,5- diphenyltetrazolium bromide (MTT-Sigma Aldrich) assay. Ninety-six-well plates precultured with test cell lines were treated with double fold serially diluted captopril and BTX-A at 37 °C for the required time interval. Post incubation period, the plates were washed twice with phosphate buffered saline (PBS) then inoculated with sterile MTT dye (0.5 mg/mL PBS) and incubated at 37 °C for 4 h. MTT was discarded and the plates were PBS washed three times. DMSO was added as 50 µL/well and the plates were shaked on plate shaker for 30 min to dissolve the produced intracellular blue formazan crystals. Optical densities (ODs) were measured at 570 nm using an ELISA plate reader (Dynatech medical products-England). Data were recorded for three independent experiments. Percentages of cell survival were calculated according to the following equation: cell survival (%) = (absorbance of treated cells/absorbance of untreated cells) X100. Half inhibitory concentration (IC_50_) was calculated depending upon the dose-response curve obtained by plotting the percentage of cell survival *versus* the concentration of tested drugs using prism program (11). Morphological changes were also observed in DU145 and HCT116 cultured plates 24 h post treatment using an inverted phase contrast microscope (Olympus-Japan) compared to untreated cells. 

*Anti-proliferation assay*

The anti-proliferative effect of BTX-A and captopril was evaluated using trypan blue dye exclusion assay, where DU145 and HCT116 cancer cell lines were plated in 24-well cell culture plates at 10^4^ cells/well and incubated at 37 °C for 24 h. At subconfluency, the plates were treated in triplicate wells with test drugs diluted in media for an additional 24 h excluding negative control wells that were inoculated with media without drugs. At the end of incubation period, the plates were washed with cold PBS. Also, the cells were detached out of the plate by trypsinization and stained with 0.4% trypan blue dye (Sigma-Aldrich-USA). The stained cells were counted using haemocytometer (New Power-Germany) as dead cells; however unstained cells were considered viable. Average count of treated and untreated wells was calculated and the data were recorded for three independent tests (12).

*Cell migration assay*

The ability of test drugs to inhibit migration of cancer cells was examined using wound scratch migration assay, where a line was drawn on the lower part of 24-well cell culture plate to divide each well into 2 equal parts. DU145 and HCT116 cancer cells were seeded in all plates and allowed to form monolayers at 80% confluency then subjected to serum starvation for 2 h. The cell monolayer was wound scratched on the previously drawn line using 200 µL sterile tip. 

The plates were washed with fresh media to get rid of the detached cells and treated with variable concentrations of test drugs for 24 h at 37 °C. Images were taken using inverted microscope to determine the wound healing in treated wells post 48 h incubation (13). Effect on cellular migration was expressed as the percentage of migration inhibition in treated cells relative to the total cell-free area in untreated cells using ImageJ software (14). 

*Effect of captopril and BTxA on expression levels of apoptosis related gene*

*RNA extraction*

RNA was extracted from Captopril (0.63, 1.25 and 2.5 mg/mL) and BTX-A (2.5, 5 and 10 U/mL) treated DU145 and HCT116 cancer cells post 24 h treatment as well as untreated cells using GeneJET RNA Purification Kit (Thermo Scientific) according to manufacturer’s procedure. The cells were collected by trypsinization, cold PBS washed twice and transferred to microcentrifuge tube. Collected cells were resuspended in 600 μL of Lysis buffer supplemented with β-mercaptoethanol. Absolute ethanol was added as 360 μL and mixed with each sample by pipetting then 700 μL of lysate were transferred to GeneJET RNA purification column inserted in a collection tube. The columns were centrifuged at 14000 rpm for 1 min, the flow-through solution was discarded and this step was repeated until all of the lysate has been transferred into the collection tube. Wash buffer 1 was added as 700 μL to the column and centrifuged as previous followed by washing twice with wash buffer 2 to ensure removal of RNA impurities. Finally, RNA was eluted using 100 µL nuclease free water and stored at -70 °C.

*Quantitative Real time PCR*

The level of expression of p53 gene was evaluated by the aid of SensiFAST SYBR Hi-ROX One-Step Kit (Bioline), where the concentration of the extracted RNA was evaluated by determining its optical density at 260 nm. Ten ng of the extracted RNA was mixed with 10 μL SensiFAST SYBR Hi-ROX One-Step Mix, forward and reverse primers (0.8 µL-10 μM), 0.2 μL reverse transcriptase, 0.4 μL RiboSafe RNase Inhibitor and the reaction mixture was completed to final volume of 20 μL using nuclease free water. Thermocycling conditions were 10 min at 45 °C for reverse transcription followed by 2 min at 95 °C for polymerase activation, then 40 cycles of denaturation (95 °C for 15 sec), annealing (55 °C for 30 sec) and extension (72 °C for 30 sec). The expression of P53 (pro-apoptotic) gene (F: 5′-TCA GAT CCT AGC GTC GAG CCC-3′ and R: 5′′-GGG TGT GGA ATC AAC CCA CAG-3′′) was normalized to the expression of β-actin gene (F: 5′-CTG GCA CCC AGC ACA ATG-3′ and R: 5′-GCC GAT CCA CAC GGA GTA CT-3′) as a housekeeping gene. Analysis of melting curve was carried out to examine the amplification specificity. Product identity was verified using agarose gel electrophoresis (15). The results were expressed as the ratio of mRNA level of p53 normalized to that of β-actin in drug treated cells compared to the untreated cells. All tests were carried out in triplicate.

*Statistical analysis*

For all experiments, the results were presented as the mean ± standard deviation of three independent experiments. Statistical significance was determined using one-way analysis of variance (ANOVA) using SPSS software. The results were considered significant at *P*-values less than 0.05.

## Results

*Determination of cytotoxicity and morphological changes*

**Figure 1:**
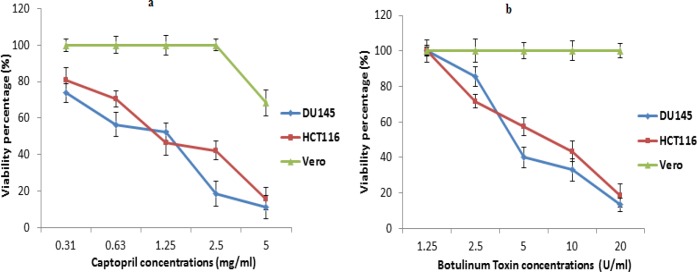
(a) Cytotoxic effect of captopril against colon cancer (HCT116), prostate cancer (DU145) and normal (vero) cell lines using MTT assay based on the amount of lactate dehydrogenase released from remaining viable cells. (b) Evaluation of cytotoxic activity of botulinum toxin Type A to cancer cell lines compared to normal cells revealing a statistically significant decrease in cellular viability in cancer cells along with increasing drug concentrations with no cytotoxic effect to vero cells at *P *< 0.05. The data were presented as mean viability percentage obtained 24 h post treatment with different concentrations ± SD

The degree of cytotoxicity of captopril and BTX-A to each cell line was determined using MTT assay, where recorded data revealed that captopril was highly cytotoxic to cancer cells with IC_50_ values of 1.2 and 1.5 mg/mL for DU145 and HCT116 cells, respectively. On the other hand, captopril was toxic to normal vero cells at only a concentration of 5 mg/mL showing 68.4% cellular viability with no toxicity at other concentrations (Figure 1a). In the mean time, BTX-A exhibited cytotoxicity to both DU145 and HCT116 cells and recording IC_50_ values of 6.4 and 7.2 U/mL respectively without significant cellular toxicity to vero cells (Figure 1b).

**Figure 2:**
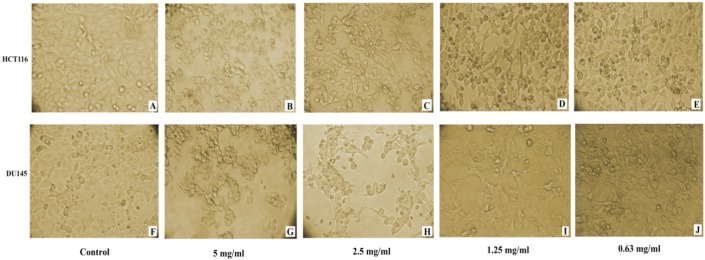
Induced cytotoxic effect in HCT116 and DU145 cancer cells 24 h post captopril treatment with different concentrations. (A and F): untreated control HCT116 and DU145; (B and G): HCT116 and DU145 cells treated with 5 mg/mL; (C and H): HCT116 and DU145 cells treated with 2.5 mg/mL; (D and I): HCT116 and DU145 cells treated with 1.25 mg/mL; (E and J): HCT116 and DU145 cells treated with 0.63 mg/mL respectively. A distinct cytotoxic effect of captopril was obvious where the cells became more dense and granulated along with increasing drug concentration

**Figure 3:**
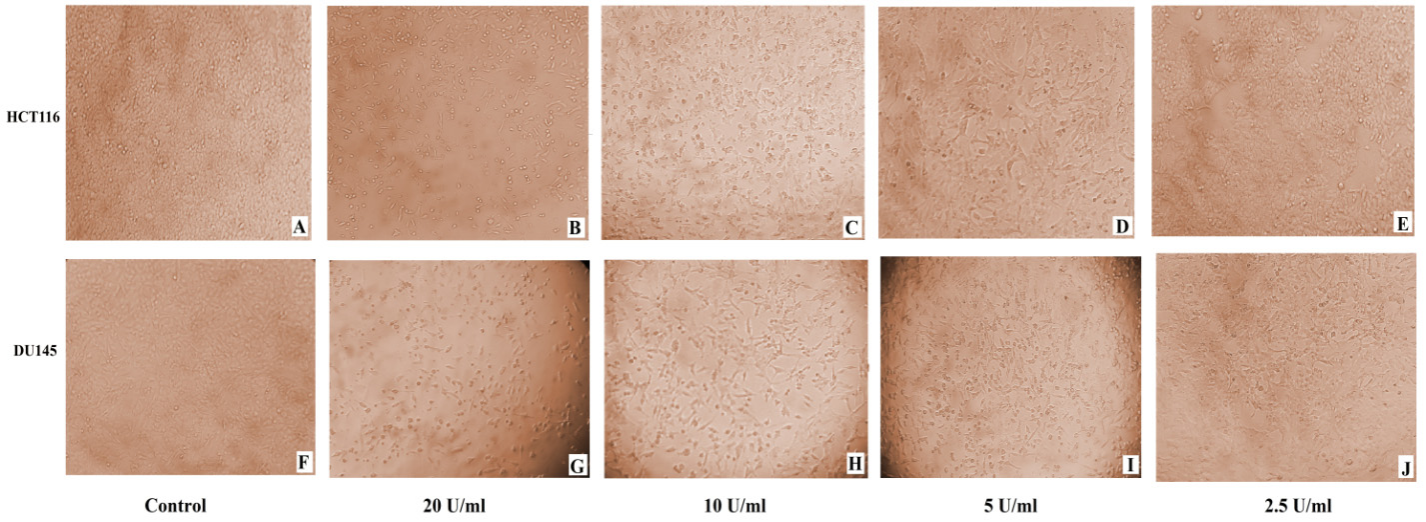
(A-J): Monitoring of morphological changes of HCT116 and DU145 cancer cells 24 h post treatment with BTxA at varible concentrations. (A and F): untreated control HCT116 and DU145; (B and G): HCT116 and DU145 cells treated with 20 U/mL; (C and H): HCT116 and DU145 cells treated with 10 U/mL; (D and I): HCT116 and DU145 cells treated with 5 U/mL; (E and J): HCT116 and DU145 cells treated with 2.5 U/mL respectively. BTxA treated cells exhibited cytotoxicity signs and detached out of the monolayer with larger areas devoid of cells at higher drug concentrations compared to normal appearance of untreated cells

Microscopic examination of DU145 and HCT116 cancer cell lines revealed cytotoxic features post 24 h treatment, where captopril and BTX-A treated cells became rounded, granulated with condensation of cellular contents and the cells become detached out of the monolayer along with increasing drug concentrations compared to the normal appearance of untreated cells (Figures 2 and 3).

*Anti-proliferation assay*

The ability of test drugs to counteract the proliferation of DU145 and HCT116 cells was examined using trypan blue dye exclusion method. 

**Figure 4:**
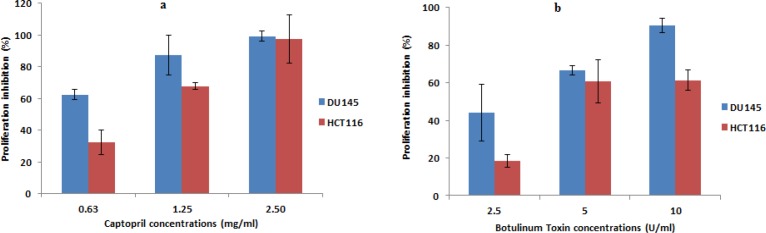
Anti-proliferative effect of captopril and BTX-A in both DU145 and HCT116 cells using trypan blue exclusion method. Recorded data revealed a statistically significant (*P *< 0.01) and a concentration dependent decrease in the cellular proliferation along with increasing drug concentration

The results showed a concentration dependent and obvious reduction in the number of unstained cells (viable cells) in captopril and BTX-A treated wells compared to negative control wells of untreated cells that were allowed to proliferate. Prostate cancer cells treated with captopril exhibited 62.3, 87.5 and 99.3 percentage inhibition post treatment at a concentration of 0.63, 1.25 and 2.5 mg/mL, respectively. While, lower inhibitory percentage was observed towards colon cancer cells by 32.5, 67.8, and 97.3%. In the same time, the anti-proliferative effect of BTX-A was apparent in both cell lines and recorded an inhibitory percentage in the order of 44.2, 66.7, 90.4%, and 18.4, 60.8, 61.4%, for DU145 and HCT116 respectively. Data was presented as percentage inhibition in the number of viable cells obtained 24 h post drug treatment (Figure 4).

*Cell migration assay*

The effect of captopril and BTX-A on the migratory potential of prostate and colon cancer cells was carried out using wound scratch migration assay. 

**Figure 5:**
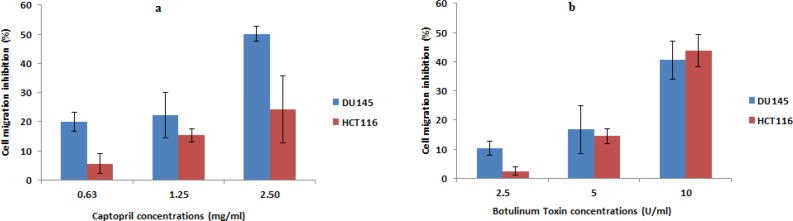
Anti-migratory effect of captopril and BTxA treated Du145 and HCT116 cells demonstrating failure of cancer cells to migrate to the damaged area in presence of test drugs

Images captured post 48 h exposure demonstrated that captopril and BTX-A could inhibit the migration of DU145 and HCT116 cells to the scratched area in a concentration dependent manner, where higher concentrations of captopril (2.5 mg/mL) induced 50.1% and 24.2% inhibition in cellular migration, respectively. In the mean time, cell migration failure was recorded upon BTX-A (10 U/mL) treatment in the order of 40.6% and 43.7% post Du145 and HCT116 treatment, respectively. Lower concentrations of both drugs resulted in a statistically significant (*P* < 0.05) inhibition in cellular migration compared to the size of scratched area in control untreated cells (Figure 5).

*Effect of captopril and BTxA on expression levels of apoptosis related gene*

**Figure 6:**
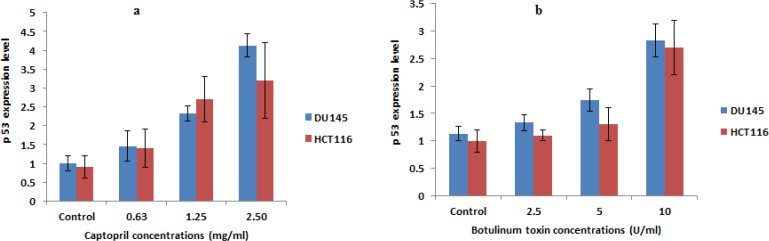
Alteration of mRNA expression of pro-apoptotic gene (p53) in DU145 and HCT116 cells post 24 h treatment with different concentrations of captopril (2.5, 1.25 and 0.63 mg/mL) and botulinum toxin type A (10, 2.5 and 1.25 U/mL) demonstrating a statistically significant (*P *< 0.01) up-regulation of p53 expression level compared to negative control cells in a dose dependent manner. Results were the mean expression levels of three independent experiments ± SD

Quantitative determination of p53 expression level indicated that captopril could potentially induce apoptosis through up-regulation of p53 gene. Captopril treated DU145 cells at concentrations of 2.5 and 1.25 mg/mL increased p53 expression level by 4 and 2.3 folds, respectively. An obvious increase in p53 expression by 2.5 and 1.5 folds was also recorded post 24 h incubation in presence of BTX-A as 10 and 5 U/mL, respectively compared to their expression levels in untreated control cells. In the mean time, a statistically significant lower elevation in the apoptotic stimulating potential was recorded in HCT116 cells post captopril and BTX-A treatment (Figure 6).

## Discussion

Carcinogenesis is a sequence of steps that are usually accompanied by abnormal cellular proliferation due to either a faulty activation of proto-oncogenes or inactivation of tumor suppressor genes. P53 is a tumor suppressor gene that is considered as the guardian of human genome as it has an essential role in disrupting the growth of cancer cells and interfering with angiogenesis (16). The success of chemotherapy or radiation therapy in killing cancer cells is hindered by obstacles. One of these obstacles is related to the numerous adverse effects caused by this type of therapy due to their cytotoxicity and apoptosis induction in normal cells such as bone marrow, lymphoid organs, hair follicles, and epithelium lining of the small intestine (17). Thus, the discovery of an alternative therapy to the currently used anti-cancer drugs has been considered as one of the most essential concerns. This was mainly to overcome their induced side effects such as myelosuppression, anemia, hair loss and most importantly due to the existence of resistance to these treatments (18). 

Overcoming the adverse effects on normal cells could be carried out through targeting p53 activation in cancer cells either through transcription-dependent or transcription-independent, but mitochondria-dependent mechanisms (19). However, the main problem in this type of therapy is that restoring the apoptotic stimulating activity of p53 gene is inhibited as a result of mutation in p53 gene which is considered as the most commonly mutated gene in human cancer (20). It was also demonstrated that regression of mutant p53 depletes the malignant properties of cancer cells, while the existence of mutation in p53 resulted in appearance of exaggerated properties in cancer cells such as metastasis and drug resistance (21). It was also reported that retaining the apoptotic stimulating activity of p53 gene could be carried out through the delivery of wild type p53 gene into cancer cells via viral targeted gene therapy (22). Another study demonstrated that the use of certain residue peptides could stabilize the tumor suppressor gene through specific binding to it (23). In the same context, a class of proteins known as mouse double minute 2 homolog (MDM2) are considered negative regulator of the p53 tumor suppressor gene (24). Thus, reactivation of p53 could be carried out by designing inhibitors of MDM2-p53 interaction, where it was demonstrated that MDM2 inhibitors could induce apoptosis and sensitize lung cancer cells to chemotherapy (25, 26). Therefore, current study aimed to evaluate captopril and BTxA as anti-cancer agents in relation to their p53 pro-apoptotic potential in cancer cells as the success of restoring the activity of p53 reported to be a miracle weapon against cancer (27).

The decreased risk for the development of prostate cancer in patients treated with captopril as an antihypertensive drug attracted the attention of researchers to investigate the anti-cancer potential of captopril (28). In the current study, captopril was investigated for its anti-cancer activity as it is available in pure form with identified structure, FDA approved and with known side effects and its pharmacokinetics have been studied extensively (9, 29). In the mean time, botulinum toxin type A is a commercially available pure pharmaceutical product with a known protein sequence (30), side effects (31) as well as toxicity and clinical studies (32). Hence, evaluating the anti-cancer efficacy of captopril and BTX-A is of great value rather than the searching for completely new drugs. DU145 is androgen-independent prostate cancer cell line that is characterized by the presence of 2 mutations in p53 gene (33). To the best of our knowledge, the *in-vitro* anti-cancer potentials of captopril and BTX-A against colon cancer cell lines has not been previously reported, thus prostate and colon cancer cells were selected as cancer cell models. 

The observed anti-cancer potential in the present study was matching other studies despite variable conditions among different studies, where *in-vivo* efficacy of captopril was demonstrated in counteracting the development of azoxymethane induced colonic premalignant lesions in diabetic and hypertensive rats (34). In addition, artesunate and captopril exhibited synergistic anti-angiogenic effect *in-vitro* and *in-vivo* (2). In the mean context, the recorded anti-cancer activity of botulinum toxin was in agreement with other studies which demonstrated the anti-cancer potential of other bacterial toxins. It was found that Coley′s toxin, which is a mixture of supernatants of *Streptococcus pyogenes* and *Serratia marcescens* cultures, was supposed to exhibit anti-cancer activity due to its ability to induce the release of tumor necrosis factor-α (TNF-α). In addition, the low molecular weight and hydrophobicity of theses bacterial peptides are important factors in their penetration into cancer cells which showed surface characters differ from that of normal cells (35). Another study observed the cytotoxic potential of recombinant diphtheria toxin (DT385) against 15 cancer cell lines due to its ability to stimulate apoptosis as well as its inhibitory effect on protein synthesis in cancer cells. Moreover, DT385 showed an anti-angiogenic effect and reduced the tumor development in chick chorioallantoic membrane. In the mean time, the subcutaneous growth of Lewis lung carcinoma tumors was significantly inhibited in animal models post DT385 treatment (36). 

Current findings revealed that BTX-A could induce cytotoxicity in cancer cell lines with an IC_50_ values of 7.2 and 6.4 U/mL for HCT116 and DU145 cells, respectively, without any toxicity to normal vero cells. In agreement with the present study, cytotoxic activity of BTX-A was recorded against human breast cancer (T47D) cells, with an IC_50_ value of 5.3 U post 24 h treatment, in a time and concentration dependent manner. However, no effect on cellular viability was reported upon treatment with lower concentrations of BTX-A as low as 0.1 U. In the same time, greater cytotoxicity to T47D cells was observed compared to normal MCF10A cells (11). Differences in IC_50_ values between both studies could be justified by the variation among cell lines in the response to BTX-A. Also, it is worth to point out that the success of the novel cancer therapy doesn’t rely only on the cytotoxicity to cancer cells, but the specific toxicity to cancer cells in association with lower toxicity to normal cells should be taken into consideration (37). Therefore, the currently recorded low cytotoxicity as well as the safety of captopril and BTX-A to normal vero cells, respectively, highlights the significant potentials of both drugs in minimizing the undesirable side effects on normal cells. In addition, the marked differences in cytotoxicity between cancer and vero cells could be rationalized by the variation between cancer and normal cells in the surface antigenic structure (35).

Regarding anti-proliferative effect of captopril and BTX-A, the results showed suppression in cellular proliferation of cancer cells which was directly proportional to the increase in the concentrations of the tested drugs. In the mean context, a study reported that BTX-A reduces cellular growth and proliferation of both LNCaP and PC-3 prostate cell lines as well as in cells transformed by phospholipase C-Gamma 1 overexpression (38, 39). While another study demonstrated that BTX-A inhibits the proliferation and showed an apoptotic effect to prostate cancer (LNCaP) but not to PC-3 cells (40). On the contrary to the previously recorded findings, captopril didn’t show anti-proliferative effect on human mammary ductal carcinoma cells except in presence of sub-physiologic concentrations of copper salts (41). In another study, captopril showed no anti-proliferative effect on breast tumor (MDA-MB-361), melanoma (Fem-x), cervical carcinoma (HeLa), and human myelogenous leukemia (K562) cell lines (42). This was also justified by the reported clinical evidence of the differences in frequencies of polymorphisms within many of cancer drug-related genes which subsequently lead to variable response to anti-cancer agents (43). 

One of the major problems in treatment of cancer is the metastasis of cancer cells. Metastasis is defined as the ability of cancer cells to invade other tissues and divide inside it in uncontrolled manner. Thus, searching an effective alternative therapy to manage the problem of metastasis is of great concern, especially that conventional therapies were unsuccessful (38). Captopril anti-metastatic potential that was recorded in the current study against colon and prostate cancer cell lines was in consistence with another study which reported that captopril could reduce the growth of colorectal cancer liver metastases *in-vivo *(44). Moreover, the anti-metastatic activity of captopril was recorded against human lung cancer (LNM35) cells which were injected in mice (45). However, the anti-metastatic activity of BTxA was seldom reported. 

P53 is a tumor suppressor gene that is activated in response to cellular stress or DNA damage and directed to induce cellular arrest in cancer cells. As a consequence to this cellular stress, p53 stimulates apoptosis via two major apoptosis-initiating pathways, designated as death receptor (extrinsic) and mitochondria-dependent (intrinsic) apoptotic pathways. The extrinsic signaling pathway that initiates apoptosis involves transmembrane receptor-mediated interactions. These involve death receptors that were members of the tumor necrosis factor (TNF) receptor. Members of TNF receptor have a cytoplasmic domain called the “death domain”. This death domain plays a critical role in transmitting the death signal from the cell surface to the intracellular signaling pathways resulting in apoptosis (46). 

The intrinsic signaling pathway that initiates apoptosis involves non-receptor-mediated stimuli releasing intracellular signals that act directly on targets within the cell. These stimuli cause changes in the inner mitochondrial membrane resulting in an opening of the mitochondrial permeability transition (MPT) pore and loss of the mitochondrial transmembrane potential accompanied with release of two main groups of pro-apoptotic proteins from the intermembrane space into the cytosol. The first group of pro-apoptotic proteins is proteins that activate the caspase dependent mitochondrial pathway including cytochrome c, Smac/DIABLO, and the serine protease HtrA2/Omi. While, the second group of pro-apoptotic proteins, namely, AIF (Apoptosis Inducing Factor), endonuclease G (Endo G) and caspase-activated DNase (CAD), are released from the mitochondria during apoptosis causing DNA fragmentation and condensation of peripheral nuclear chromatin. It is essential to focus on that the control and regulation of these apoptotic mitochondrial events occurs through members of Bcl-2 family proteins. In addition, the tumor suppressor protein, p53, has a critical role in the regulation of these proteins (47). 

Depending on the fact that p53 plays an essential role in the determining the fate of cancer cells, the therapeutic strategies that target the activation of p53-mediated apoptotic pathway could present a novel and effective pathway to destruct cancer cells (48). Consequently, quantitation of the effect of captopril and BTxA on the expression level of a pro-apoptotic (p53) gene was performed in the present study to find out the relation between the recorded anti-cancer activities of tested drugs and their apoptotic stimulating potentials. Moreover, real time PCR was selected for evaluating the expression level as it is the most reliable and accurate method (49). Current results revealed that the anti-cancer activity of tested drugs is related to their up-regulation potential of p53 gene which resulted in apoptosis induction. In consistence with these findings, it was reported that the cytotoxic effect of BTX-A on breast cancer cells was associated with apoptosis induction (11). Moreover, the efficacy of injecting botulinum neurotoxin Type A was recently demonstrated in reducing the lower urinary tract symptoms accompanied by benign prostatic hyperplasia due to enhancement of apoptosis (50). Also, captopril exhibited an *in-vivo* apoptotic stimulating activity against human lung cancer (LNM35) cells post injection in mice (45).

Accordingly, the use of captopril in regulating the blood pressure in cancer patients could be beneficial in preventing the progress of their cases. Hypertension was also recorded to be a common risk factor for chemotherapy, thus captopril could be combined with chemotherapeutic agents to make use of its anticancer and antihypertensive effects (51). In addition, there is no fear from development of cancer associated with the use of BTX-A either in cosmetic or therapeutic applications. The cytotoxic and anti-proliferative as well as the anti-metastatic efficacy of captopril and BTX-A indicated that both drugs could present a novel and potential therapeutic strategy in cancer therapy through stimulating the intrinsic pathway of apoptosis. Also, restoring p53 apoptotic potential by the effect of captopril and BTX-A may play a role in overcoming the problem of resistance to anti-cancer drugs through directing cancer cells to self-destruction with minimal effect on the neighboring normal tissues.
